# Characterisation of Genome-Wide PLZF/RARA Target Genes

**DOI:** 10.1371/journal.pone.0024176

**Published:** 2011-09-20

**Authors:** Salvatore Spicuglia, Christelle Vincent-Fabert, Touati Benoukraf, Guillaume Tibéri, Andrew J. Saurin, Joaquin Zacarias-Cabeza, David Grimwade, Ken Mills, Boris Calmels, François Bertucci, Michael Sieweke, Pierre Ferrier, Estelle Duprez

**Affiliations:** 1 Centre d'Immunologie de Marseille-Luminy (CIML), Université de la Méditerranée, Campus de Luminy, Marseille, France; 2 Institut National de la Santé et de la Recherche Médicale (INSERM) U631, Marseille, France; 3 Centre National de la Recherche Scientifique (CNRS), UMR 6102, Marseille, France; 4 Institut National de la Santé et de la Recherche Médicale (INSERM) U891, Centre de Recherche en Cancérologie de Marseille, Marseille, France; 5 Institut Paoli-Calmettes, Marseille, France; 6 Institut de Biologie du Développement de Marseille Luminy, Université de la Méditerranée, Campus de Luminy, Marseille, France; 7 Centre National de la Recherche Scientifique (CNRS), UMR 6216, Marseille, France; 8 Department of Medical and Molecular Genetics, King's College London School of Medicine, London, United Kingdom; 9 Centre for Cancer Research and Cell Biology, Queen's University Belfast, Belfast, United Kingdom; 10 Cancer Science Institute of Singapore, National University of Singapore, Singapore, Singapore; 11 Oncologie Moléculaire, Institut Paoli-Calmettes, Marseille, France; Institute of Genetics and Molecular and Cellular Biology, France

## Abstract

The PLZF/RARA fusion protein generated by the t(11;17)(q23;q21) translocation in acute promyelocytic leukaemia (APL) is believed to act as an oncogenic transcriptional regulator recruiting epigenetic factors to genes important for its transforming potential. However, molecular mechanisms associated with PLZF/RARA-dependent leukaemogenesis still remain unclear.

We searched for specific PLZF/RARA target genes by ChIP-on-chip in the haematopoietic cell line U937 conditionally expressing PLZF/RARA. By comparing bound regions found in U937 cells expressing endogenous PLZF with PLZF/RARA-induced U937 cells, we isolated specific PLZF/RARA target gene promoters. We next analysed gene expression profiles of our identified target genes in PLZF/RARA APL patients and analysed DNA sequences and epigenetic modification at PLZF/RARA binding sites. We identify 413 specific PLZF/RARA target genes including a number encoding transcription factors involved in the regulation of haematopoiesis. Among these genes, 22 were significantly down regulated in primary PLZF/RARA APL cells. In addition, repressed PLZF/RARA target genes were associated with increased levels of H3K27me3 and decreased levels of H3K9K14ac. Finally, sequence analysis of PLZF/RARA bound sequences reveals the presence of both consensus and degenerated RAREs as well as enrichment for tissue-specific transcription factor motifs, highlighting the complexity of targeting fusion protein to chromatin. Our study suggests that PLZF/RARA directly targets genes important for haematopoietic development and supports the notion that PLZF/RARA acts mainly as an epigenetic regulator of its direct target genes.

## Introduction

Retinoic acid receptors (RARs) belong to a family of nuclear receptors that can activate or repress transcription of target genes by recruiting co-activator or co-repressor complexes. It is the binding of its natural ligand, retinoic acid (RA), which transforms the activity of RAR from a repressor to an activator of transcription by inducing a conformational change in the ligand binding domain structure [1], [2]. In acute promyelocytic leukaemia (APL), the RAR alpha (*RARA*) gene on chromosome 17, finds itself involved in chromosomal recombination with one of several different potential partner genes (designated “X”), i.e. *PML*, *PLZF*, *NuMA*, *NPM1*, *FIP1L1*, *PRKAR1A*, *STAT5b* or *BCOR*, generating X/RARA fusion proteins [3], [4]. Evidence to date suggests that X/RARA induces a characteristic block in differentiation, associated with the APL phenotype through repression of downstream target genes [1]–[4]. The capacity of X/RARAs to form homo-oligomers and hetero-oligomers has been proven critical for inducing transformation and defines their oncogenic propensity [5]–[8]. However, it is still not well understood the extent to which the X/RARA protein, as part of an oligomeric complex, has acquired altered DNA-binding capacity that may result in aberrant expression of genes normally (or not) regulated by wild-type RARA. Several studies have suggested that the presence of the partner X enforces a different conformation of the DNA recognition site for X/RARA versus RARA and that X/RARA would recognise degenerate RA response elements [8]–[10]. This phenomenon could be amplified by recruitment of co-factors that may also impose and/or change the DNA recognition specificity [11].

The nature of the fusion partner X has a decisive impact on the disease state and sensitivity to the therapeutic effects of *all-trans* RA (ATRA) and arsenic trioxide (ATO) [12]. Importantly, PLZF/RARA-associated APL is resistant to ATO and exhibits impaired sensitivity to ATRA, giving rise to a significantly poorer clinical outcome compared to patients with classical PML-RARA+ disease, which typically responds to both of these molecularly-targeted therapies [13], [14]. Studies conducted over a decade ago provided insights into the distinct natural history of these subtypes of APL, showing major differences in the capacity of the PML/RARA and PLZF/RARA fusion proteins to bind corepressor complexes according to the level of retinoic acid. While corepressors are displaced from the PML/RARA oncoprotein in the presence of pharmacological doses of retinoid, binding of SMRT/NCoR-HDAC [15], [16] and epigenetic factors such as Polycomb group (PcG) complexes [17] to PLZF/RARA persists under such conditions through interaction with the PLZF moiety of the fusion protein [18]. These specific recruitments provide an explanation for the ATRA-resistant phenotype and suggest there may be mechanistic differences in transcriptional repression mediated by PLZF/RARA as compared to other X/RARA fusions. Due to the PLZF moiety, PLZF/RARA could potentially control two different sets of genes: those genes normally regulated by RARA-RXR and those that are *de novo* target genes due to the sequence-specific DNA binding activity of PLZF. In addition, the situation is made more complicated by expression of the reciprocal RARA/PLZF that can potentially bind to PLZF binding sites where it may function as a transcriptional activator [19]. The development of genome-wide approaches such as ChIP-on-chip has favoured the identification of genomic targets that have widened our understanding of oncogenic transcription factor activities.

Here, we report a genome-wide ChIP-on-chip study of PLZF/RARA gene targets using an inducible cell system. We identify 413 high-confidence specific target genes that provide clues as to how PLZF/RARA contributes to APL pathogenesis.

## Results and Discussion

### Genome-wide identification of specific PLZF/RARA target genes

**Figure 1:**
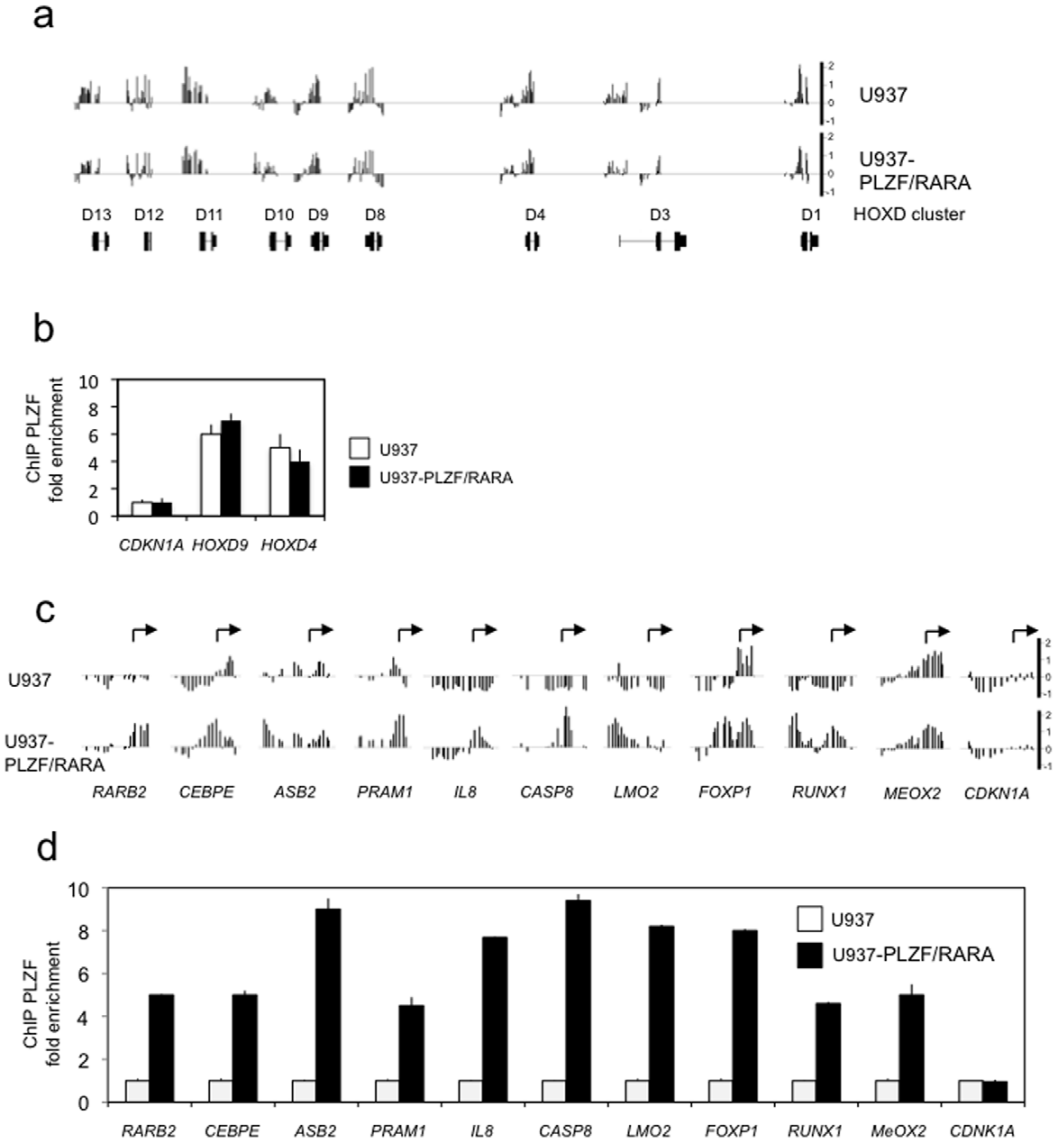
Genome-wide identification of PLZF/RARA-binding sites using ChIP-on-chip (a) ChIP-on-chip profiles of PLZF(/RARA) binding along the *HOXD* gene cluster on chromosome 2 in U937 and U937-PLZF/RARA cell lines as indicated in the figure. The plots show the log2 ratio of PLZF ChIP over the INPUT, combined from two independent experiments. Positions of the genes are shown under the profiles. (b) ChIP-qPCR PLZF fold enrichment on *CDKN1A*, *HOXD9* and *HOXD4* promoters in the absence or in the presence of PLZF/RARA. (c) Zoomed-in view of PLZF binding at 11 selected promoters as indicated, showing differences upon PLZF/RARA induction. (d) PLZF/RARA fold enrichment was measured for the selected promoters by ChIP-qPCR in PLZF/RARA expressing cells.

To identify gene targets directly regulated by PLZF/RARA, we took advantage of a zinc-inducible U937 cell system either expressing the PLZF/RARA fusion protein or harbouring an empty vector [20]. In the U937-B412 cell line, PLZF/RARA expression can be induced upon zinc induction (Figure S1). We performed ChIP experiments using an anti-PLZF antibody that recognises both wild-type PLZF and the PLZF/RARA fusion protein. Immunoprecipitated DNA samples were hybridised to a custom promoter array containing ∼18,000 human promoters and replicate experiments were merged (see Materials and Methods). Using a peak detection algorithm [21] we identified a total of 1545 significantly bound promoter regions in the U937-PLZF/RARA cell line. Among these targets, we found previously characterised PLZF targets, such as several genes from the *HOXD* cluster [17], [22] (Figure 1a). Indeed, we detected high enrichment signals at the promoter regions of the *HOXD4* and *HOXD9* genes in both U937-PLZF/RARA and control cell lines. These data were corroborated by quantitative PCR analyses of independent PLZF ChIP samples (ChIP-qPCR) (Figure 1b). To identify PLZF/RARA-specific target genes we compared the targets found in the zinc-stimulated U937-PLZF/RARA cell line to those found in the zinc-stimulated control U937-empty vector cell line and identified 412 promoter regions that were significantly bound only upon PLZF/RARA induction (Table S1). Among these genes, we identified *RARB2* (the RARA fusion model target gene) as well as *C/EBPE*, *ASB2*, *PRAM1* and *IL8*, that have all previously been reported to be regulated by X/RARA fusion proteins [23]–[26]. To further validate the specificity of our approach, we performed quantitative PCR analyses on ChIP samples (ChIP-qPCR) on a selection of ten PLZF/RARA specific target genes and one negative control (Fig. 1c). All tested PLZF/RARA–bound promoter regions were significantly amplified in the induced U937-PLZF/RARA cell line only (Fig. 1d), underscoring the specificity of the generated PLZF/RARA-binding profiles.

In a recent study, Rice and colleagues [26] identified more than 4900 genes as direct PLZF and/or PLZF/RARA targets. However, in this work the PLZF and PLZF/RARA targets were not discriminated, making it difficult to compare with the specific PLZF/RARA targets identified in the present study. Nevertheless, 168 specific PLZF/RARA target genes from our study were also identify as potential targets in the Rice study. The discrepancies between the two studies might be explained by the different experimental procedures and by the methods and the thresholds used to select *bona-fide* binding regions. Functional classification of specific PLZF/RARA target genes according to gene ontology (GO) analyses showed that PLZF/RARA binds to genes implicated in either basic cellular processes or developmental related processes (Table 1). Strikingly, a significant number of the gene products are involved in the regulation of transcription, highlighting the oncogenic potential that could arise from their deregulation, including transcription factors implicated in haematopoietic development, such as C/EBPA, C/EBPE, GATA1 and BCL6, as well as other general chromatin modifiers such as BRD2, MBD6 and SMARCD2 (Table 2).

**Table 1:** Gene ontology analysis of PLZF/RARA direct target genes.

Biological process	Count	%	P-Value
Signal transduction	80	23.70%	1.70e-05
Cell motility	24	7.10%	5.50e-05
Protein phosphorylation	50	14.80%	6.50e-05
mRNA transcription regulation	153	45.30%	1.40e-04
Fatty acid beta-oxidation	10	3.00%	1.80e-04
mRNA transcription	101	29.90%	1.30e-03
Protein modification	53	15.70%	1.70e-03
Cell proliferation and differentiation	26	7.70%	2.60e-03

**Table 2:** PLZF/RARA selected target genes.

Regulation of transcription		
CEBPE	CCAAT/enhancer binding protein epsilon	**RARE**
RARB	Retinoic acid receptor, beta	**RARE**
TRIM38	Tripartite motif-containing 38	**RARE**
TBX20	T-box 20	
CEBPA	CCAAT/enhancer binding protein (C/EBP), alpha	
RGS12	Regulator of G-protein signaling 12	**RARE**
BIN1	Bridging integrator 1	
EGLN2	Egl nine homolog 2 (C. elegans)	**RARE**
GTPBP3	GTP binding protein 3 (mitochondrial)	**RARE**
DDIT3	DNA-damage-inducible transcript 3	**RARE**
FOS	V-fos FBJ osteosarcoma viral oncogene homolog	
MEOX2	Mesenchyme homeobox 2	
BCL3	B-cell CLL/lymphoma 3	**RARE**
GATA1	GATA binding protein 1	**RARE**
CDKN1C	Cyclin-dependent kinase inhibitor 1C (p57, Kip2)	
BHLHB2	Basic helix-loop-helix domain containing class B 2	**RARE**
BCL6	B-cell CLL/lymphoma 6 (zinc finger protein 51)	
BCL11A	B-cell CLL/lymphoma 11A (zinc finger protein)	**RARE**
LMO2	LIM domain only 2 (rhombotin-like 1)	
CSDA	Cold shock domain protein A	**RARE**
FOXP1	Forkhead box P1	**RARE**
SKIL	SKI-like oncogene	**RARE**
SMARCD2	SWI/SNF related, subfamily d, member 2	**RARE**
ETV5	Ets variant gene 5 (ets-related molecule)	**RARE**
FHL1	Four and a half LIM domains 1	
POU4F3	POU class 4 homeobox 3	
AIRE	Autoimmune regulator	
LPXN	Leupaxin	**RARE**
CDCA7L	Cell division cycle associated 7-like	**RARE**
BRD2	Bromodomain containing 2	**RARE**
ZFP91	Zinc finger protein 91 homolog (mouse)	**RARE**
ETV1	Ets variant gene 1	**RARE**
RORC	RAR-related orphan receptor C	**RARE**
SOX12	SRY (sex determining region Y)-box 12	**RARE**
POU3F3	POU class 3 homeobox 3	
MBD6	Methyl-CpG binding domain protein 6	**RARE**

Neuron development/cell morphogenesis		
MAP1B	Microtubule-associated protein 1B	
LST1	Leukocyte specific transcript 1	
AXUD1	AXIN1 up-regulated 1	**RARE**
NGRN	Neugrin, neurite outgrowth associated	**RARE**

Signal transduction		
ARHGAP12	Rho GTPase activating protein 12	
PLEKHH2	Pleckstrin domain containing, family H member 2	**RARE**
GAB1	GRB2-associated binding protein 1	
STAP2	Signal transducing adaptor family member 2	**RARE**
OSBPL8	Oxysterol binding protein-like 8	
PRAM1	PML-RARA regulated adaptor molecule 1	**RARE**
IL8	Interleukin 8	
TGFB1	Transforming growth factor, beta 1	

Post-translational protein modification		
CDKN1C	Cyclin-dependent kinase inhibitor 1C (p57, Kip2)	
CDK5	Cyclin-dependent kinase 5	**RARE**
ASB2	Ankyrin repeat and SOCS box-containing 2	**RARE**
UBE4B	Ubiquitination factor E4B (UFD2 homolog, yeast)	**RARE**
DAPK3	Death-associated protein kinase 3	
ITPKB	Inositol 1,4,5-trisphosphate 3-kinase B	
PFKP	Phosphofructokinase, platelet	**RARE**
SNRK	SNF related kinase	**RARE**
MAP3K8	Mitogen-activated protein kinase kinase kinase 8	**RARE**
PKN2	Protein kinase N2	
CASP8	Caspase 8, apoptosis-related cysteine peptidase	
TBL1XR1	Transducin (beta)-like 1X-linked receptor 1	
HECW1	E3 ubiquitin protein ligase 1	**RARE**
RBKS	RIBOKINASE	**RARE**
DUSP4	Dual specificity phosphatase 4	**RARE**
DUSP6	Dual specificity phosphatase 6	**RARE**
PLCB2	Phospholipase C, beta 2	**RARE**
PLCD3	Phospholipase C, delta 3	

Mitochondrion		
PDP2	Pyruvate dehydrogenase phosphatase isoenzyme 2	**RARE**
MRPL54	Mitochondrial ribosomal protein L54	**RARE**
MTRF1L	Mitochondrial translational release factor 1-like	**RARE**
NDUFA4	NADH dehydrogenase 1 alpha subcomplex, 4, 9 kDa	**RARE**
MTX1	Metaxin 1	**RARE**
Magmas	Mitochondria-associated protein	

Integral/Intrinsic to Membrane		
DHRS3	Dehydrogenase/reductase (SDR family) member 3	**RARE**
CD38	CD38 molecule	**RARE**
IL21R	Interleukin 21 receptor	**RARE**
GPR81	G protein-coupled receptor 81	
SERPINE1	Serpin peptidase inhibitor, clade E	**RARE**
SERPINB6	Serpin peptidase inhibitor, clade B, member 6	
ICAM1	Intercellular adhesion molecule 1 (CD54)	**RARE**
THBS3	Thrombospondin 3	**RARE**
TCIRG1	T-cell, immune regulator 1, lysosomal V0 subunit A3	**RARE**
KAZALD1	Kazal-type serine peptidase inhibitor domain 1	**RARE**
DLL3	Delta-like 3 (Drosophila)	
DLL4	Delta-like 4 (Drosophila)	
SYT3	Synaptotagmin III	**RARE**
SCAMP4	Secretory carrier membrane protein 4	**RARE**
MOXD1	Monooxygenase, DBH-like 1	
ITGB2	Integrin beta 2 (CD18)	**RARE**
LTB	Lymphotoxin beta (TNF superfamily, member 3)	**RARE**
AGTRAP	Angiotensin II receptor-associated protein	**RARE**
IL4I1	Interleukin 4 Induced 1	**RARE**
FLOT1	Flotillin 1	**RARE**
MAN1C1	Mannosidase, alpha, class 1C, member 1	**RARE**
TNFSF4	Tumor necrosis factor member 4	**RARE**
MEGF11	Multiple EGF-like-domains 11	
FAM69A	FAMILY WITH SEQUENCE SIMILARITY 69	**RARE**
HIGD1A	HIG1 domain family, member 1A	**RARE**

### Gene expression changes at PLZF/RARA target genes

**Figure 2:**
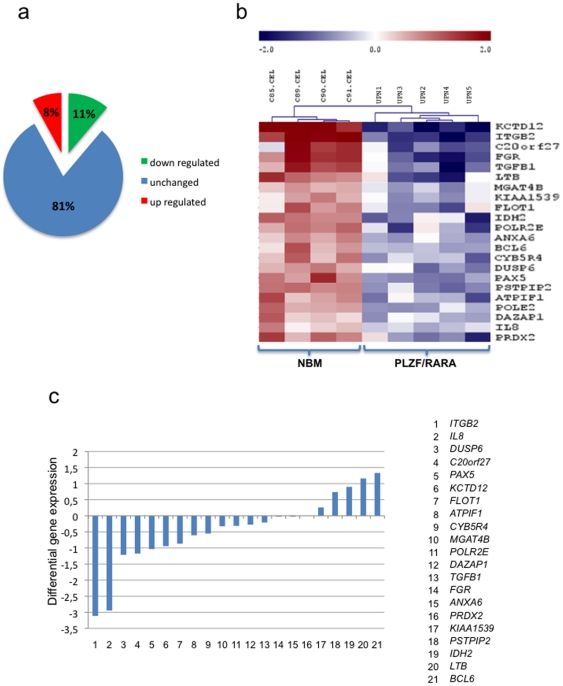
Expression profile of PLZF/RARA target genes. (a) Analysis of variations in PLZF/RARA-direct target gene expression after PLZF/RARA induction in the B412 cell line. (b) Heat map representing mRNA expression levels of PLZF/RARA-direct target genes in PLZF/RARA positive APL samples and normal bone marrow (NBM) samples. Relative expression levels are normalized across the samples; levels greater than or less than the mean are shown in shades of red and blue, respectively. (c) Changes in expression of the 23 genes identified in (b) in response to PLZF/RARA induction in U937-B412 cells. The differential gene expression corresponds to the difference of microarray fluorescence intensity between MT and B412 cell lines for the indicated genes.

To determine the transcriptional effect of PLZF/RARA on its identified target genes, gene expression profiling was performed in U937-MT (control) and U937-B412 (PLZF/RARA) cell lines. Expression profiling following PLZF/RARA induction was evaluated by microarray analysis and 4321 genes were identified with altered expression levels (>1.5 fold; *P*<0.05). A highly significant 20% of the genes targeted by PLZF/RARA have altered expression patterns (*P* = 4.99×10^−9^, *Fisher exact*) with 11% becoming down regulated and 9% being up regulated upon PLZF/RARA binding (Figure 2a). This result is consistent with previous microarray analyses that have revealed that PLZF/RARA induces a signature characterised by both down regulated and up regulated genes [24], [26]. This suggests that while PLZF/RARA can act as a transcriptional regulator, effects of PLZF/RARA binding to chromatin cannot be measured only by transcriptional analysis. In addition, this shows that the direct transcriptional activity of PLZF/RARA is difficult to assess in an established cell line that has already shut down transcriptional pathways. To analyze the significance of PLZF/RARA targets identified by ChIP-on-chip with the APL phenotype we performed analyses of microarray gene-expression profiles from diagnostic samples from PLZF/RARA+ patients. These analyses identified 22 direct PLZF/RARA target genes that were significantly down regulated in PLZF/RARA APL blasts implicating their deregulation in tumour pathogenesis (Figure 2b). The analysis confirmed that the *ITGB2* gene encoding CD18, which is characteristically under expressed in primary APL cells (a feature used to help identify patients with PML-RARA+ disease) [27], was also down regulated in the presence of the PLZF/RARA fusion. Moreover, we demonstrated that several PLZF/RARA-target genes relevant for malignant haematopoiesis were significantly down regulated in PLZF/RARA patient samples, including *IDH2*, *PAX5* and *DAZAP1*. *IDH2* is frequently mutated in acute myeloid leukaemia and its mutations confer adverse prognosis in cytogenetically normal AML [28], [29]. *PAX5* and *DAZAP1* are both involved in translocations associated with acute lymphoblastic leukaemia [30], [31]. In addition, PLZF/RARA significantly down regulated genes involved in self-renewal. This is the case for transforming growth factor beta-1 (TGFbeta-1), a pluripotent cytokine that controls key tumour suppressive functions. Interestingly, cytoplasmic PML has been described as a critical TGF-beta regulator and PML/RARA APL cells have defects in TGF-beta signalling similar to those observed in PML-null cells [32], [33]. This is also the case for DUSP6, an inhibitor of the ERK signalling that has been described to be under expressed in different types of cancer, including pancreatic [34] and lung cancer [35], and which was reported as a potential PLZF-RARA target gene by Rice and colleagues [26].

The expression profiles obtained with the patient samples correlates with the expression profile obtained in B412 cell system as the majority of the genes down regulated in PLZF/RARA+ patients were also down regulated in B412 cells expressing PLZF/RARA (Figure 2c). This integrated genome wide analysis thus highlights the importance of a subset of PLZF/RARA target genes and reveals new pathways that could be disrupted in the PLZF/RARA APL.

### Chromatin modification at PLZF/RARA target genes

**Figure 3:**
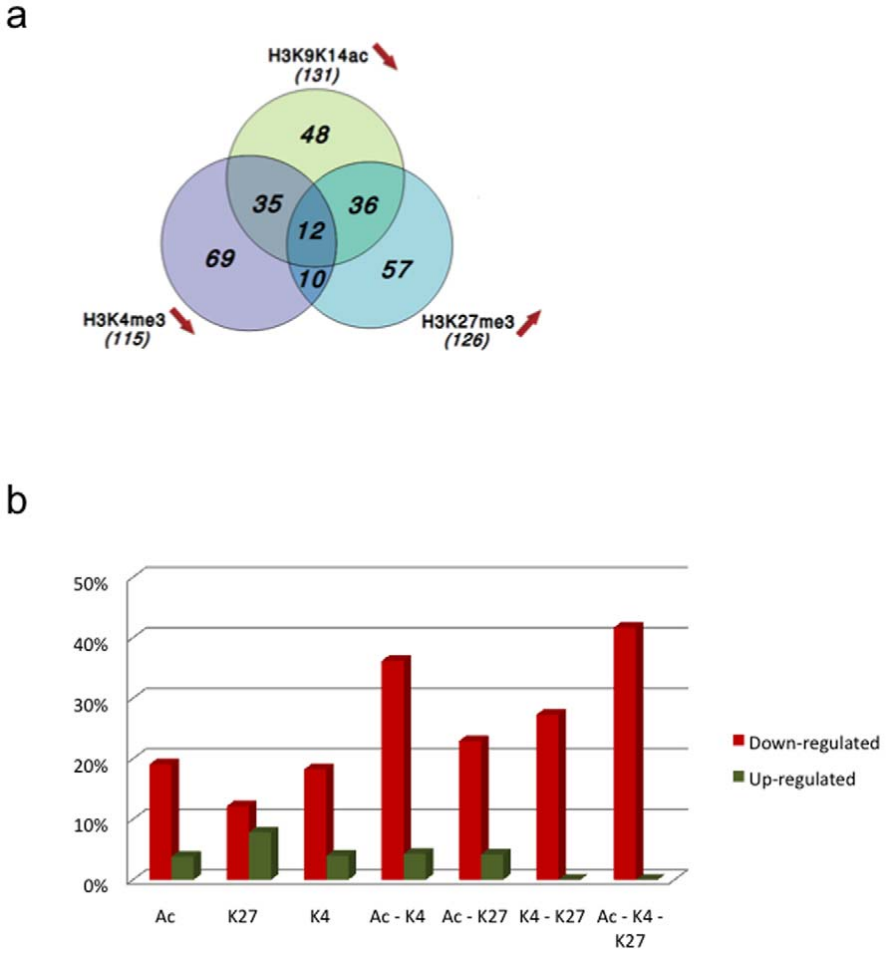
Chromatin modifications at PLZF/RARA target genes. (a) Venn diagram representing the combinatorial changes of histone modifications at PLZF/RARA target genes. The total number of PLZF/RARA target gene promoters displaying decreased levels of H3K14ac or H3K4me3, or increased levels of H3K27me3 is also indicated. (b) The bar plot indicates the percentages of either repressed or induced genes, accordingly to the combinatorial changes of histone modifications. The percentages are relative to gene numbers indicated in Figure 3a.

PLZF/RARA was shown to act as a dominant-negative inhibitor of RARA, mediating repression of retinoid target genes important for myeloid differentiation: PLZF/RARA binding to *RARB2* has previously been shown to recruit PcG complexes, leading to an increase in trimethylation of Lysine 27 of histone H3 (H3K27me3) and *RARB2* repression (ref. [17] and data not shown). To investigate whether PLZF/RARA can induce chromatin modification at its target genes, we analysed the trimethylation status of lysines 4 and 27 of histone H3 (H3K4me3 and H3K27me3) and the acetylation status of histone H3 (H3K9K14Ac) in response to PLZF/RARA-induction by ChIP-on-chip. Indeed, PLZF/RARA induction provokes epigenetic variations represented as an increase in H3K27me3, a decrease in H3K4me3 or a decrease in H3K9K14Ac on 267 of its 413 target genes (Figure 3a). To evaluate the consequences of epigenetic changes on gene expression, we calculated the percentages of deregulated genes in function of individual or combinatorial changes for each histone modification (Figure 3b). We observed that the correlation between histone modifications and gene expression changes was higher when taking into account at least 2 of the analysed marks. Strikingly, genes displaying epigenetic changes for all three of the histone modifications displayed the higher percentage of repressed genes. These results strongly support the idea that PLZF/RARA protein could recruit different co-repressor complexes to its target gene promoters, resulting in a multiplicity of epigenetic changes.

**Figure 4:**
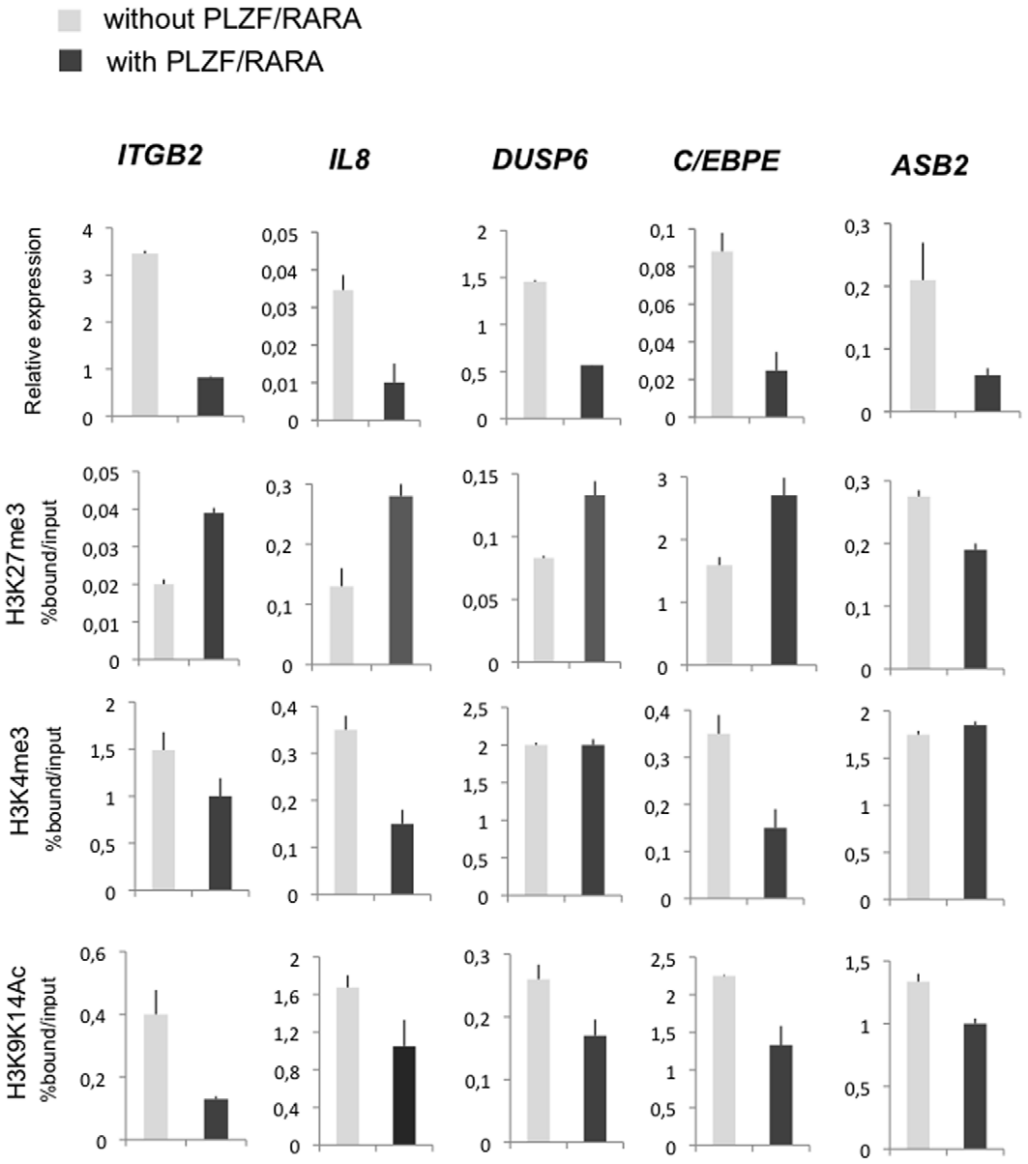
Validation of gene expression and chromatin modification changes at PLZF/RARA target genes. Zinc-induced U937-MT cells (light grey bars) or U937-B412 cells (dark grey bars) were analysed as follows. For mRNA expression level, RNA was extracted and reverse-transcribed; the selected target genes were analyzed by qRT-PCR. Gene expression of each gene is shown relative to *HPRT* expression. For epigenetic marks, H3K27me3, H3K4me3 and H3Ac were analysed by ChIP-qPCR at the indicated promoter.

To validate the transcriptional and epigenetic effects of PLZF/RARA on its identified target genes, we analysed the mRNA expression level as well as changes in epigenetic histone modifications of a subset of PLZF/RARA targets. The five selected genes were repressed by direct PLZF/RARA binding, while their epigenetic changes were showing similar characteristics (Figure 4). With the exception of *ASB2*, the genes down regulated by PLZF/RARA recruitment display an increase in H3K27me3 at their promoters with a concomitant decrease in H3K9K14Ac (Figure 4). H3K4me3 was either decreased or unmodified but was not found increased at PLZF/RARA repressed loci (Figure 4). Interestingly, *ITGB2* and *IL8* that are strongly repressed in B412 cell line and that display epigenetic changes for the 3 histone modifications (Figure 4), were down regulated in PLZF/RARA+ patient (Figure 2). These observations emphasize the importance of these two genes in APL pathology and highlight the relevance of epigenetic changes consistent with a PcG-repressed chromatin state that is mediated by PLZF/RARA binding.

In this study we show that PLZF/RARA expression induces epigenetic changes in a subset of genes of which expression is not necessarily modified. This observation is interesting in the context of the different nature of the fusion partner X that impact on the therapeutic effects of ATRA and ATO. Previous studies have demonstrated that X/RARA proteins recruit different partners that exert influences upon disease characteristics with respect to sensitivity to RA treatment [15]–[18]. In this study we reveal an epigenetic profile that is characteristic to PLZF/RARA bound loci. While a study has shown that high levels of H3K27me3 was associated with the PML/RARA binding region in the *RARB2* promoter [36], genome-wide analysis of H3K27me3 at PML/RARA binding sites reveals no significant increase of this repressive mark related to PML/RARA induction [3]. Thus, characterisation of epigenetic changes induced by X/RARA, even if not associated with detectable changes in gene expression, could provide an explanation for the difference in sensitivity to RA.

### Sequence analysis of the PLZF/RARA-bound DNA

**Figure 5:**
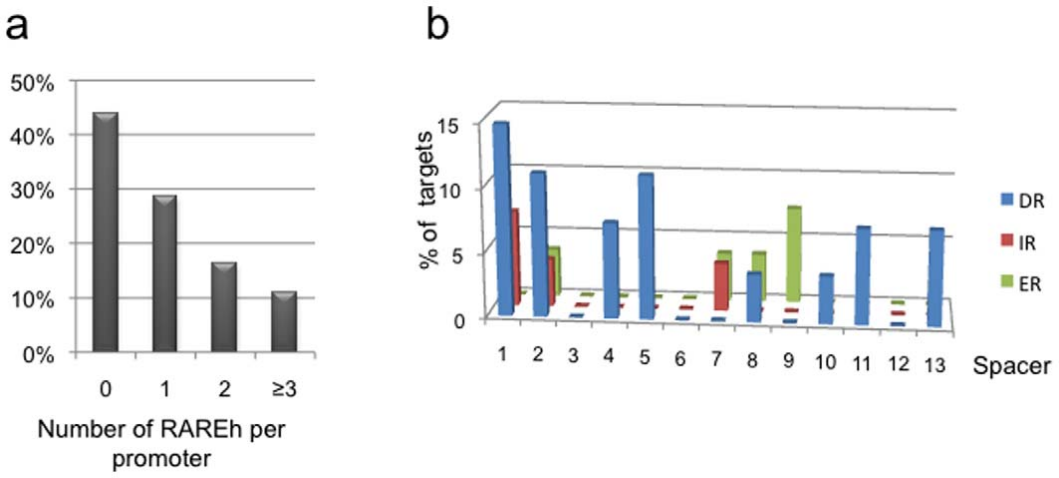
Presence of RARE in PLZF/RARA target promoters. (**a**) Percentage of PLZF/RARA bound regions containing 0, 1, 2, and 3 or more the core motif RGKTCA or RARE half site (RAREh). (**b**) PLZF/RARA target regions were scanned for the presence of Direct (DR), Inverse (IR) or Everted (ER) RARE repeats with a maximum spacer length of 13 nucleotides. The % of target promoters as a function of spacer length is plotted.

PLZF/RARA has conserved the DNA binding domain of RARA that binds to RA response elements (RAREs) defined by a direct repeat of the core motif 5′-RGKTCA-3′ separated by 1, 2 or 5 nucleotides (DR1, DR2, DR5) [9]. To determine whether the core motif RGKTCA (RARE half-site or RAREh) was the determining factor in recruiting PLZF/RARA, the sequences overlapping each peak were analyzed for the presence of this motif. We found that approximately half (233 out of 412) of the PLZF/RARA-bound DNA sequences contained at least one RAREh and that among these DNA sequences, almost 50% of them contained two or more repeats of the RAREh (Figure 5a). Spacing and orientation of core motifs dictate the specificity of DNA recognition by the nuclear receptors and the X/RARA fusion proteins [37]. We analysed for the presence of the possible combination of the core motif sequence; direct repeat (DR), inverse repeat (IR) or everted repeat (ER) in PLZF/RARA promoters where we could detect more than one RAREh. This showed that binding more frequently occurs at DR elements than at IR or ER motifs, with DR1, DR2 and DR5 accounting for 35% of promoter sites targeted by PLZF/RARA overall (Figure 5b). Thus, PLZF/RARA appears to bind to promoter regions harbouring either consensus or highly degenerated RARE, but the presence of RAREs is not absolutely required for PLZF/RARA to bind to its target promoters. However, we could not find a correlation between the presence of RARE in PLZF/RARA binding sites and PLZF/RARA target gene expression (Table S1). This result confirms previous observations suggesting that the binding of X/RARA to chromatin does not require direct consensus RARE [37]. This is due to the presence of the fusion partner that could extend or modify the range of accessible DNA response elements beyond those bound by native RARA [39]. The binding of PLZF/RARA to promoter regions in the absence of RAREh suggests that other factors influence PLZF/RARA chromatin targeting. For example, chromatin structure could influence PLZF/RARA targeting. A recent genome-wide study showed that even in the case of wild-type RARA, DR1, DR2 or DR5 elements do not account for the majority of bound loci, proposing that chromatin topology contributes to RAR occupancy [38]. A potential difference between PLZF as a fusion partner and most of the other APL fusion partners (e.g. PML) is that PLZF can bind DNA in its own right. Although the PLZF DNA binding domain is disrupted by the fusion, through heterodimerization PLZF/RARA could be targeted to PLZF binding sites. In order to find out whether significant binding sites can be identified in PLZF/RARA bound regions we searched for transcription factor (TF) binding sites using Genomatix (Table 3 and Figure S2). We found that PLZF/RARA target regions were significantly enriched for several TF binding sites, including the KLF and ETS family of TFs. However, we did not find that PLZF/RARA target regions were specifically enriched for PLZF binding sites. Interestingly, PML/RARA was recently shown to be targeted to genes regulated by PU.1, a member of the ETS family [4]. Thus, it is possible that like PML/RARA, PLZF/RARA collaborates with other tissue-specific TFs for DNA binding.

**Table 3:** Transcription factor matrix families over-represented in PLZF/RARA target regions*.

TF Families	Description	Number of Sequences	Overepresentation (promoters)	Z-Score (promoters)
V$KLFS	Krueppel like transcription factors	327	1,71	21,17
V$ETSF	Human and murine ETS1 factors	327	1,23	6,51
V$EGRF	EGR/nerve growth factor induced protein C & related factors	321	2,22	34,33
V$ZBPF	Zinc binding protein factors	318	2,23	36,84
V$SP1F	GC-Box factors SP1/GC	312	2,08	28,06
V$CTCF	CTCF and BORIS gene family	305	2,3	29,94
V$RXRF	RXR heterodimer binding sites	286	1,19	4,76

*The most frequent matrix families are shown.

### Conclusion

By comparing PLZF bound regions between U937 and PLZF/RARA-induced U937 cells we identified 413 specific PLZF/RARA target genes. This selection of bona-fide PLZF/RARA targets was significantly enriched in genes important for hematopoietic development and included genes effectively downregulated in primary PLZF/RARA+ APL cells. Moreover, PLZF/RARA binding was preferentially associated with gene repression at sites enriched for the repressive H3K27me3 histone modification mark. Analysis of PLZF/RARA bound sequences suggests PLZF/RARA binds canonical and degenerate RARE motifs, and recruitment of PLZF/RARA may also occur via other hematopoietic specific TFs. This study helps to clarify the role of the PLZF/RARA fusion protein as an oncogenic transcriptional regulator during leukaemogenesis and provides further insights into the pathogenesis of the disease.

## Materials and Methods

### Cell lines

Human myelomonoblastic cell lines U937-MT and U937-B412 [20] were maintained at exponential growth in RPMI supplemented with 10% foetal calf serum. U937-MT is the empty vector control, U937-B412 contains PLZF/RARA cDNA under the control of the zinc inducible human-metallothionein promoter [20]. For PLZF/RARA induction cells were prestimulated with 0.1 mM ZnSO_4_ for at least 24 h.

### RNA isolation and RT-PCR

Total RNA was extracted using the RNeasy Mini kit according to the manufacturer's protocol (Qiagen), For cDNA synthesis, reverse transcription was performed with 2 µg of the total RNA, oligo dT, dNTPs, DTT, buffer and Superscript Retrotranscriptase II (Invitrogen). cDNA was analyzed by qPCR using Brilliant SYBR Green Master Mix (Stratagene) according to the manufacturer's instructions with the 7500 Fast Real Time PCR system (Applied Biosystems). Primers used for real-time PCR are given in Supporting Information.

### ChIP

Chromatin Immunoprecipitation (ChIP) was essentially performed as described before by Boukarabila et al. [17]. U937 Cells were treated with 1% formaldehyde (RT, 10 min), and chromatin was extracted then sonicated for 15 min using a Diagenode Bioruptor (full power, 30 s on 30 s off). For ChIP using PLZF antibody, (H300; Santa Cruz), chromatin extracted from 1×10^7^ cells per condition was used. For ChIP using H3K27me3 (Abcam, ab6002), H3K4me3 (Diagenode, pAB-003-050) or H3K9K14Ac (Millipore, 06-599), chromatin from 5×10^6^ cells per condition was used. Chromatin was diluted 5× in 150 mM NaCl, 10 mM Tris-HCl pH 8.0, 1% TritonX-100, 0.1% sodium deoxycholate (NaDOC), 1 mM EDTA, 0.5 mM EGTA. Chromatin, precleared 1 h with protein G-coupled magnetic beads, was incubated overnight at 4°C using the different antibodies followed by an incubation for 2 h with blocked protein G-coupled magnetic beads (Dynabeads, Invitrogen). Real-time PCR was performed using Brilliant SYBR Green Master Mix (Stratagene) according to the manufacturer's instructions with the 7500 Fast Real Time PCR system (Applied Biosystems). Primers used are given in Text S1.

### ChIP-on-chip

Total DNA and ChIP DNA were amplified using the whole genome amplification kit (WGA-2, Sigma-Aldrich, Taufkirchen, Germany) and fluorescently labelled using BioPrime Array CGH Genomic labelling kit (Invitrogen). Labeled samples were hybridized following the manufacturer's instructions to a custom promoter array (Agilent, Santa Clara, USA) containing 236.992 probes spanning the promoter regions (−2,5 kb to +1 kb) of 18.000 refseq genes (UCSC assembly HG17). Experiments were performed in duplicate and showed high correlation (R≥0.769). All data is MIAME compliant. Median-normalized log2 enrichment ratios (ChIP/Input) of merged replicates were calculated using CoCAS software [21]. For PLZF ChIP-on-chip experiments, significantly enriched promoters were isolated using the peak detection algorithm in CoCAS and the following settings: 2× and 1× standard deviations for main and extended thresholds, respectively. Enrichment scores were calculated by measuring the effective peak area [21]. For histone modification experiments, enrichment scores were calculated by computing the average signals at each promoter region. Next, fold changes between MT and B412 chromatin at PLZF/RARA target promoters were calculated for each epigenetic mark. Gene promoters displaying a differential enrichment of >0,135 (P val<0.05) for a given histone modification, were considered to be differentially marked.

### Functional annotation of genes

Target genes were classified using DAVID [39] and according to PANTHER molecular functions and biological process categories. Only the highest significant categories were selected (P<0,001).

### Sequence analysis

We looked for the consensus RARE motif using Perl regular expressions (http://www.perl.org) on ChIP-on-chip peak sequences. The expression code was built according to the known repeat pattern (A/G)G(G/T)TCA. We allowed a gap ranging from 0 to 100 bases and performed the calculation for finding DR, IR and ER on both strands. Analyses of transcription factor binding site enrichments were performed using MatInspector tool from GENOMATIX software (http://www.genomatix.de).

### Microarray analysis

MT and B412 cells were cultured in duplicate in the presence of zinc for 48 hours. RNA was extracted using QIAGEN RNeasy and quality was tested using an Agilent Bioanalyzer. 1 µg of RNA was hybridized to U133 Plus 2.0 arrays (Affymetrix). Affymetrix U133A.CEL data files from B412 cells and MT cells were imported into R and probe intensities were normalized using GC-RMA. A total of 4321 genes were found to be differentially expressed (>1,5 fold; *P*<0.05). For patient samples, microarray raw data was taken from Gene Expression Omnibus (http://www.ncbi.nlm.nih.gov/projects/geo/), under the data set GSE8510 for primary PLZF/RARA+ APL patient cases and under the data set GSE15061 for normal bone marrow samples (NBM). Details of the patient samples analysed have been reported previously [19]. Expression data were Robust Multichip Average (RMA) [40] normalised using the ‘affy’ R/Bioconductor library. The differential gene expression study was performed by Significance Analysis of Microarrays using MeV software [41].

## Supporting Information

Figure S1 **Expression of PLZF/RARA, PLZF and RARA proteins in MT and B412 cell lines.** Nuclear extracts were prepared from U937-MT (1) and U937-B412 (2) treated with zinc and were immunobloted for PLZF/RARA and PLZF (α-PLZF) or RARA (α-RARA).
(TIF)

Figure S2 **Transcription factor binding sites over-represented in PLZF/RARA target promoters.** MatInspector tool from GENOMATIX was used to identified transcription binding sites in PLZF/RARA bound promoters.
(TIF)

Table S1 **List of PLZF/RARA target genes.** Indicated in the table are symbols and full names of PLZF/RARA target genes, the presence, number and type of RAREh in PLZF/RARA enriched peak and micro array data of PLZF/RARA target genes. Diff mean corresponds to the difference of the mean between MT and B412 cell lines.
(XLSB)

Text S1 **Supplemental experimental procedures.**
(DOCX)

## References

[1] Scaglioni PP, Pandolfi PP (2007). The theory of APL revisited.. Curr Top Microbiol Immunol.

[2] Hoemme C, Peerzada A, Behre G, Wang Y, McClelland M (2008). Chromatin modifications induced by PML-RARalpha repress critical targets in leukemogenesis as analyzed by ChIP-Chip.. Blood.

[3] Martens JH, Brinkman AB, Simmer F, Francoijs KJ, Nebbioso A (2010). PML-RARalpha/RXR Alters the Epigenetic Landscape in Acute Promyelocytic Leukemia.. Cancer Cell.

[4] Wang K, Wang P, Shi J, Zhu X, He M (2010). PML/RARalpha targets promoter regions containing PU.1 consensus and RARE half sites in acute promyelocytic leukemia.. Cancer Cell.

[5] Salomoni P, Pandolfi PP (2000). Transcriptional regulation of cellular transformation.. Nat Med.

[6] Kwok C, Zeisig BB, Dong S, So CW (2006). Forced homo-oligomerization of RARalpha leads to transformation of primary hematopoietic cells.. Cancer Cell.

[7] Sternsdorf T, Phan VT, Maunakea ML, Ocampo CB, Sohal J (2006). Forced retinoic acid receptor alpha homodimers prime mice for APL-like leukemia.. Cancer Cell.

[8] Zhou J, Peres L, Honore N, Nasr R, Zhu J (2006). Dimerization-induced corepressor binding and relaxed DNA-binding specificity are critical for PML/RARA-induced immortalization.. Proc Natl Acad Sci U S A.

[9] Hauksdottir H, Privalsky ML (2001). DNA recognition by the aberrant retinoic acid receptors implicated in human acute promyelocytic leukemia.. Cell Growth Differ.

[10] Jeanne M, Lallemand-Breitenbach V, Ferhi O, Koken M, Le Bras M (2010). PML/RARA oxidation and arsenic binding initiate the antileukemia response of As2O3.. Cancer Cell.

[11] Zhu J, Nasr R, Peres L, Riaucoux-Lormiere F, Honore N (2007). RXR is an essential component of the oncogenic PML/RARA complex in vivo.. Cancer Cell.

[12] Grimwade D, Mistry AR, Solomon E, Guidez F (2010). Acute promyelocytic leukemia: a paradigm for differentiation therapy.. Cancer Treat Res.

[13] Licht JD, Chomienne C, Goy A, Chen A, Scott AA (1995). Clinical and molecular characterization of a rare syndrome of acute promyelocytic leukemia associated with translocation (11;17).. Blood.

[14] Petrie K, Zelent A, Waxman S (2009). Differentiation therapy of acute myeloid leukemia: past, present and future.. Curr Opin Hematol.

[15] Grignani F, De Matteis S, Nervi C, Tomassoni L, Gelmetti V (1998). Fusion proteins of the retinoic acid receptor-alpha recruit histone deacetylase in promyelocytic leukaemia.. Nature.

[16] He LZ, Guidez F, Tribioli C, Peruzzi D, Ruthardt M (1998). Distinct interactions of PML-RARalpha and PLZF-RARalpha with co- repressors determine differential responses to RA in APL.. Nat Genet.

[17] Boukarabila H, Saurin AJ, Batsche E, Mossadegh N, van Lohuizen M (2009). The PRC1 Polycomb group complex interacts with PLZF/RARA to mediate leukemic transformation.. Genes Dev.

[18] Lin RJ, Nagy L, Inoue S, Shao W, Miller WH (1998). Role of the histone deacetylase complex in acute promyelocytic leukaemia.. Nature.

[19] Guidez F, Parks S, Wong H, Jovanovic JV, Mays A (2007). RARalpha-PLZF overcomes PLZF-mediated repression of CRABPI, contributing to retinoid resistance in t(11;17) acute promyelocytic leukemia.. Proc Natl Acad Sci U S A.

[20] Ruthardt M, Testa U, Nervi C, Ferrucci PF, Grignani F (1997). Opposite effects of the acute promyelocytic leukemia PML-retinoic acid receptor alpha (RAR alpha) and PLZF-RAR alpha fusion proteins on retinoic acid signalling.. Mol Cell Biol.

[21] Benoukraf T, Cauchy P, Fenouil R, Jeanniard A, Koch F (2009). CoCAS: a ChIP-on-chip analysis suite.. Bioinformatics.

[22] Barna M, Merghoub T, Costoya JA, Ruggero D, Branford M (2002). Plzf mediates transcriptional repression of HoxD gene expression through chromatin remodeling.. Dev Cell.

[23] Moog-Lutz C, Peterson EJ, Lutz PG, Eliason S, Cave-Riant F (2001). PRAM-1 is a novel adaptor protein regulated by retinoic acid (RA) and promyelocytic leukemia (PML)-RA receptor alpha in acute promyelocytic leukemia cells.. J Biol Chem.

[24] Park DJ, Vuong PT, de Vos S, Douer D, Koeffler HP (2003). Comparative analysis of genes regulated by PML/RAR alpha and PLZF/RAR alpha in response to retinoic acid using oligonucleotide arrays.. Blood.

[25] Guibal FC, Moog-Lutz C, Smolewski P, Di Gioia Y, Darzynkiewicz Z (2002). ASB-2 inhibits growth and promotes commitment in myeloid leukemia cells.. J Biol Chem.

[26] Rice KL, Hormaeche I, Doulatov S, Flatow JM, Grimwade D (2009). Comprehensive genomic screens identify a role for PLZF-RARalpha as a positive regulator of cell proliferation via direct regulation of c-MYC.. Blood.

[27] Paietta E (2003). Expression of cell-surface antigens in acute promyelocytic leukaemia.. Best Pract Res Clin Haematol.

[28] Abbas S, Lugthart S, Kavelaars FG, Schelen A, Koenders JE (2010). Acquired mutations in the genes encoding IDH1 and IDH2 both are recurrent aberrations in acute myeloid leukemia: prevalence and prognostic value.. Blood.

[29] Paschka P, Schlenk RF, Gaidzik VI, Habdank M, Kronke J (2010). IDH1 and IDH2 mutations are frequent genetic alterations in acute myeloid leukemia and confer adverse prognosis in cytogenetically normal acute myeloid leukemia with NPM1 mutation without FLT3 internal tandem duplication.. J Clin Oncol.

[30] Yuki Y, Imoto I, Imaizumi M, Hibi S, Kaneko Y (2004). Identification of a novel fusion gene in a pre-B acute lymphoblastic leukemia with t(1;19)(q23;p13).. Cancer Sci.

[31] O'Neil J, Look AT (2007). Mechanisms of transcription factor deregulation in lymphoid cell transformation.. Oncogene.

[32] Lin HK, Bergmann S, Pandolfi PP (2004). Cytoplasmic PML function in TGF-beta signalling.. Nature.

[33] Lin HK, Bergmann S, Pandolfi PP (2005). Deregulated TGF-beta signaling in leukemogenesis.. Oncogene.

[34] Xu S, Furukawa T, Kanai N, Sunamura M, Horii A (2005). Abrogation of DUSP6 by hypermethylation in human pancreatic cancer.. J Hum Genet.

[35] Okudela K, Yazawa T, Woo T, Sakaeda M, Ishii J (2009). Down-regulation of DUSP6 expression in lung cancer: its mechanism and potential role in carcinogenesis.. Am J Pathol.

[36] Villa R, Pasini D, Gutierrez A, Morey L, Occhionorelli M (2007). Role of the polycomb repressive complex 2 in acute promyelocytic leukemia.. Cancer Cell.

[37] Kamashev D, Vitoux D, de Thé H (2004). PML-RARA-RXR oligomers mediate retinoid and rexinoid/cAMP cross-talk in acute promyelocytic leukemia cell differentiation.. J Exp Med.

[38] Delacroix L, Moutier E, Altobelli G, Legras S, Poch O (2009). Cell-specific interaction of retinoic acid receptors with target genes in mouse embryonic fibroblasts and embryonic stem cells.. Mol Cell Biol.

[39] Huang da W, Sherman BT, Lempicki RA (2009). Systematic and integrative analysis of large gene lists using DAVID bioinformatics resources.. Nat Protoc.

[40] Bolstad BM, Irizarry RA, Astrand M, Speed TP (2003). A comparison of normalization methods for high density oligonucleotide array data based on variance and bias.. Bioinformatics.

[41] Saeed AI, Sharov V, White J, Li J, Liang W (2003). TM4: a free, open-source system for microarray data management and analysis.. Biotechniques.

