# Supplementary figures and images for: Characterisation of Genome-Wide PLZF/RARA Target Genes
Source: PLoS One. 2011 Sep 20;6(9):e24176. doi: 10.1371/journal.pone.0024176 (PMC3176768; doi:10.1371/journal.pone.0024176)

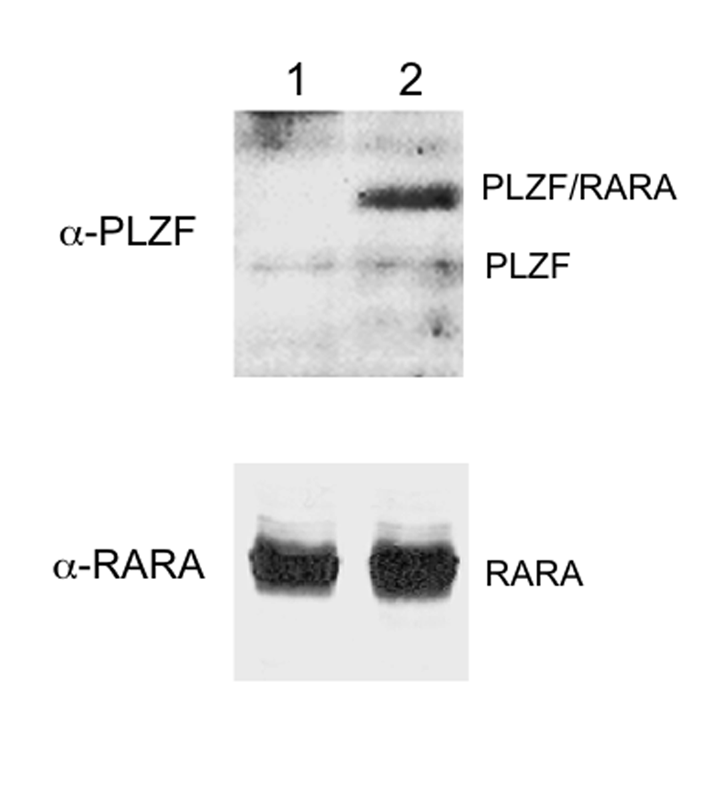

Supplement: Figure S1 — Expression of PLZF/RARA, PLZF and RARA proteins in MT and B412 cell lines. Nuclear extracts were prepared from U937-MT (1) and U937-B412 (2) treated with zinc and were immunobloted for PLZF/RARA and PLZF (α-PLZF) or RARA (α-RARA). (TIF) [file pone.0024176.s001.tif]

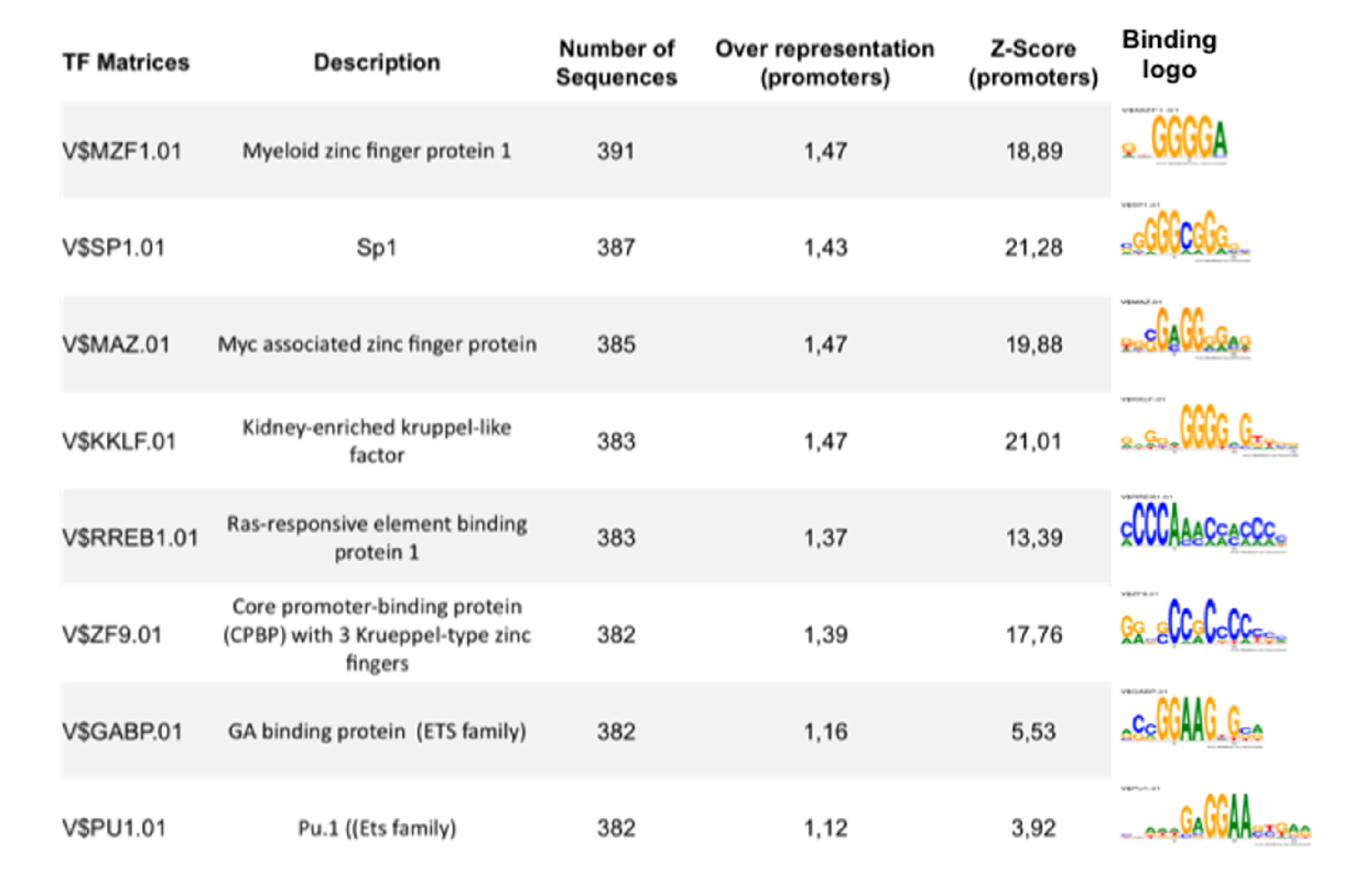

Supplement: Figure S2 — Transcription factor binding sites over-represented in PLZF/RARA target promoters. MatInspector tool from GENOMATIX was used to identified transcription binding sites in PLZF/RARA bound promoters. (TIF) [file pone.0024176.s002.tif]
