# Supplementary material for: Characterisation of Genome-Wide PLZF/RARA Target Genes
Source: PLoS One. 2011 Sep 20;6(9):e24176. doi: 10.1371/journal.pone.0024176 (PMC3176768; doi:10.1371/journal.pone.0024176)
Supplement: Text S1 — Supplemental experimental procedures. (DOCX) [file pone.0024176.s004.docx]

**Supplemental experimental procedures**

PCR Primers used for ChIP-qPCR analysis.

*ASB2*; Forward 5’CTGGGGTTCTAAGGCCATCT

Reverse 5’GGCCACAGACTTCAGTCCTC

*CASP8*; Forward 5’CCGACGGTTAAGTACTTTATTCTG

Reverse 5’CTGTCTTTGGGGAACAAGAGA

*C/EBPE*; Forward 5’CTCGATGTAGGCGGAGAGG

Reverse 5’TGGCCAGCAGCCACTCGAG

*CDKN1A*; Forward 5’ TCACAGGTGTTTCTGCGGC

Reverse 5’ CCGCGCACTTAGAGACACC

*CDKN1C*; Forward 5’ GTACTGGGAAGGTCCCACG

Reverse 5’ATGTCCGACGCGTCCCTCC

*FOXP1*; Forward 5’ GGTGTTTTCAACCCCCTTTCTC

Reverse 5’ TGGACAAAGTGCAGAAAGAAAGAG

*GATA1*; Forward 5’CAGCCCTGAATCCCTTTACA

Reverse 5’ GGGGTATAGCGAGCAAAGTG

*HOXD4*; Forward 5’ TGGTCTACCCCTGGATGAAG

Reverse 5’ TGACCTGCTCCCTCAGCTAT

*HOXD9*; Forward 5’AGCCTATACCCCAAGCCACT

Reverse 5’ CTTCCAGGTGGGCAAGACTA

*IL8*; Forward 5’ GAGCACTCCATAAGGCACAAACT

Reverse 5’ GATGGTTCCTTCCGGTGGTT

*LMO2*; Forward 5’ GTGGTCTGGTGGTTAGCATACG

Reverse 5’ TTCCCCTAAGCCCCAGAAA

*MEOX2*; Forward 5’ TGGATTCTGGGTGAAAGCAT

Reverse 5’ CAAACCAAAAGGACCGTTGT

*PRAM1*; Forward 5’ CTGCAACCACTCCTCAGA

Reverse 5’ CTTTCCAAGGAGCAATCCTG

*RARB2*; Forward 5’ TGTGAGAATCCTGGGAGTTGGTGA

Reverse 5’TGCCTCTGAACAGCTCACTTCC

*RUNX1*; Forward 5’ GCTGTGGGTTGGTGATGCT

Reverse 5’ GGACGAATCACACTGAATGCA

*TGFBETA*; Forward 5’ GGGGATCTGTGGCAGGTC

Reverse 5’ CTTCATCCCGGCTGTCTC

PCR Primers used for RT-qPCR analysis.

*ASB2*; Forward; GTATCTGCCCCAGAGGTGAG

Reverse; GTCAGCCAGAGGTCTTGGAC

*C/EBPE*; Forward; CCAAGCGCAAGAGATCAGCG

Reverse; CTCTGCCATGTACTCCAGCA

*DUSP6*; Forward; CCTGGAAGGTGGCTTCAGTA

Reverse; CGGTCAAGGTCAGACTCGAT

*IL8*; Forward; CATACTCCAAACCTTTCCACCC

Reverse; TTTCTGTGTTGGCGCAGTGT

*ITGB2*; Forward;CCTCAACGAGATCACCGAGT

Reverse; TTGTTGGTCAGCTTCAGCAC
